# Improving detection and treatment of psychological distress during menopause: evidence from a clinical hormone replacement therapy cohort

**DOI:** 10.1192/bji.2025.10094

**Published:** 2026-05

**Authors:** Olivia Hendriks, Aini Kamal, Pooja Saini, Abigail K. Rose, Daniel Reisel, Louise Newson, Jason C. McIntyre

**Affiliations:** 1 Research Fellow at the School of Psychology, Liverpool John Moores University, Liverpool, UK.; 2 Clinical Research Associate at Clinical Research Division, Newson Clinic, Stratford-Upon-Avon, UK; 3 Professor of Suicide and Self-Harm Prevention at the School of Psychology, Liverpool John Moores University, Liverpool, UK; 4 Reader in Psychology at the School of Psychology, Liverpool John Moores University, Liverpool, UK; 5 Specialist Registrar at Obstetrics and Gynaecology, University College London, London, UK; 6 GP and Menopause Specialist at Menopause & Wellbeing Service, Newson Clinic, Stratford-Upon-Avon, UK; 7 Senior Lecturer at the School of Psychology, Liverpool John Moores University, Liverpool, UK

**Keywords:** Hormone replacement therapy, menopause, depression, suicidality, testosterone

## Abstract

**Background:**

Menopausal transition is a period of psychological vulnerability, yet suicidality remains underassessed. Hormone replacement therapy (HRT) may influence mood symptoms, but its mental health effects – particularly regarding suicidality – are poorly understood.

**Aims:**

To evaluate changes in depressive symptoms, menopause-related distress and suicidality among menopausal women attending a specialist clinic, and explore whether outcomes differed across HRT regimens and baseline risk factors.

**Method:**

We analysed routinely collected data from 957 women attending a UK menopause clinic. All participants received some form of treatment following their initial consultation. Participants completed the Patient Health Questionnaire-9 (PHQ-9) and Menopause Depression Rating Scale (MENO-D) at baseline and follow-up (2–6 months later). Mixed-design analyses of variance assessed changes over time, including interaction effects for HRT type and baseline risk factors (body mass index (BMI), smoking, suicidality, antidepressant use).

**Results:**

Depressive symptoms and menopause-related psychological distress significantly declined over time (around 46% reduction on average). The largest improvements were observed among women receiving oestrogen–progesterone–testosterone combinations, although similar gains were also seen in oestrogen–progesterone and oestrogen–testosterone groups. Suicidality (PHQ-9 item 9) decreased by 92% among those with baseline ideation, but this was not moderated by HRT type. Self-worth (MENO-D item 4) also improved, but similarly showed no significant moderation by HRT regimen. Higher BMI was associated with worse baseline mental health, but did not moderate treatment outcomes.

**Conclusions:**

Combined HRT, including formulations with testosterone, was associated with substantial improvements in mental health outcomes. Suicidality was a distinct symptom profile, often underdetected by general depression scores. However, findings are exploratory and should be interpreted cautiously because of the lack of a control group, observational design and small sample sizes in some subgroups. These results highlight the need for menopause-sensitive mental health assessments and integration of psychological screening into routine menopausal care.

The menopausal transition is increasingly recognised as a period of heightened psychological vulnerability. Between 10 and 65% of women experience mental health challenges during this time, including depression and suicidality.^1–3^ Hormonal fluctuations are strongly implicated in mood instability,^4^ yet the mental health effects of menopause remain under-recognised in many clinical settings. Hormone replacement therapy (HRT) is often prescribed to manage somatic and psychological symptoms, with growing support for its role in alleviating low mood and anxiety with or without additional psychiatric support.^5–8^ However, evidence remains mixed – particularly regarding its efficacy for severe depression – prompting questions about optimal treatment combinations and individual differences in response.^8–10^ Emerging evidence suggests that the psychological impact of HRT varies depending on the type of hormone used, delivery method and individual factors.^11^ Oestrogen remains the most extensively studied and is associated with improvements in mood, cognition and stress sensitivity, particularly when administered transdermally during perimenopause.^12,13^ Testosterone may offer additional benefits for mood, motivation and energy, potentially through both direct neurochemical effects on dopamine and serotonin pathways^8,14^ and indirect improvements in vasomotor symptoms, libido and sleep quality^10,11^ – although findings are mixed and access remains limited.^14,15^ In contrast, progesterone’s effects on mental health appear more variable, with some studies suggesting anxiolytic properties and others reporting minimal or negative effects.^16,17^ These discrepancies highlight the need for more targeted research into hormone-specific and combination regimens for mood support during menopause.

Suicidality is among the most concerning, yet underinvestigated, aspects of menopause-related distress. Although midlife women have the highest suicide rates among females in the UK,^18^ their psychological symptoms are often overlooked, minimised or misattributed to general ageing or lifestyle changes.^12^ Standard tools like the Patient Health Questionnaire-9 (PHQ-9^19^) are commonly used to assess depressive symptoms, but they were not designed for the menopausal context. Crucially, they may underdetect suicidality when it is treated merely as a component of total depression scores, despite growing evidence that suicidal ideation can manifest independently.^13,20^

The Menopausal Depression Rating Scale (MENO-D^21^) was developed to address this gap by capturing menopause-specific symptoms often missed by general scales. It was validated in a midlife female cohort and demonstrated strong psychometric properties. The scale includes ten domains specific to menopausal experience, such as paranoia, libido changes, self-worth and cognitive symptoms. Importantly, the ‘self’ domain contains an item on low self-worth that, at its highest severity level, references suicidal thinking. Although not yet widely adopted, it may offer a more accurate lens on the psychological toll of menopause than general depression scales alone.

This study aimed to examine how different HRT regimens influence mental health outcomes, including depressive symptoms, menopause-related distress and suicidality, among women attending a specialist menopause and well-being clinic. We hypothesised that combined HRT regimens, especially those including oestrogen and testosterone, would be associated with greater symptom improvements than progesterone-only treatments. All participants received treatment following their consultation; therefore, there was no formal no-HRT control group. We also explored whether suicidality and self-worth would change over time and whether treatment effects varied based on baseline risk factors such as body mass index (BMI), antidepressant use and initial symptom severity. By using both general (PHQ-9) and menopause-specific (MENO-D) measures, this study sought to clarify the psychological impact of HRT and offer more tailored insights into mental healthcare during the menopausal transition.

## Method

### Design and participants

This longitudinal, observational study used routinely collected clinical data from 957 patients attending a UK-based private menopause clinic. All participants had completed at least one PHQ-9 and one MENO-D assessment at two time points (baseline and follow-up). Inclusion criteria for the analytic sample were: (a) completion of baseline (time point 1) and follow-up (time point 2) PHQ-9 and MENO-D scores; (b) prescription of systemic HRT following clinical consultation and (c) a classifiable HRT regimen based on available prescription data. Participants with missing primary outcome data or unclear HRT classification were excluded from treatment-based analyses, but retained in descriptive reporting where possible. No exclusions were made based on age, ethnicity or comorbidities. Participant characteristics are detailed in Table 1. For descriptive purposes, Table 1 compares participants based on whether they were already using HRT before their initial consultation. These pre-consultation categories do not reflect treatment groups, but rather baseline starting points to contextualise later prescribing patterns. Menopause stage and depression diagnosis were recorded during initial clinic consultations by general practitioners (GPs) based on patient self-report and clinical judgement. These diagnoses were extracted from routine clinical notes and not independently verified by the research team.

**Table 1:** Participant characteristics overall and by baseline hormone replacement therapy use (*n* = 957)

Variable	Category	*n* (%)/mean (s.d.)	HRT at baseline (*n* = 492)	No HRT at baseline (*n* = 465)
Age, years		51.21 (6.28)	51.68 (5.29)	50.70 (7.20)
Weight (kg)		72.20 (16.89)	71.94 (16.12)	72.46 (17.76)
Height (m)		1.66 (0.07)	1.66 (0.07)	1.66 (0.07)
BMI		26.26 (5.90)	26.16 (5.71)	26.36 (6.11)
Ethnicity, *n* (%)				
	White	906 (94.7)	464 (94.3)	442 (95.1)
	Black	3 (0.3)	1 (0.2)	2 (0.4)
	Asian	16 (1.7)	8 (1.6)	8 (1.7)
	Mixed/other	32 (3.3)	19 (3.9)	13 (2.8)
Alcohol use, *n* (%)	Yes	678 (70.8)	394 (80.1)	335 (72.0)
Smoking status, *n* (%)	Yes	36 (3.8)	17 (3.5)	19 (4.1)
Menopause diagnosis, *n* (%)				
	Perimenopause	459 (48.0)	196 (39.8)	263 (56.6)
	Menopause	359 (37.5)	208 (42.3)	151 (32.5)
	Other^a^	139 (14.5)	88 (17.9)	51 (11.0)
Antidepressant use	Yes	181 (18.9)	94 (19.1)	87 (18.7)
HRT use at baseline	Yes	492 (51.4)	492 (100)	0 (0.0)
Depression diagnosis	Yes	294 (30.7)	146 (29.7)	148 (31.8)

HRT, hormone replacement therapy; BMI, body mass index.

a. ‘Other’ refers to ambiguous or non-standard diagnostic entries (e.g. surgical, early or late menopause). All cases were retained in the analyses. Columns represent participant characteristics based on baseline HRT status, i.e. whether they were taking any form of HRT at the time of first clinic contact. These groupings were used to describe the sample only and do not reflect final treatment groups used in analyses.

### HRT classification

Participants were grouped into HRT treatment categories based on the combination of hormones received after their initial consultation: oestrogen, progesterone and testosterone. The most common regimens included oestrogen and progesterone (*n* = 326, 34.1%), testosterone only (*n* = 281, 29.4%) and oestrogen, progesterone and testosterone together (*n* = 210, 21.9%). Smaller groups received oestrogen only (*n* = 49, 5.1%), oestrogen and testosterone (*n* = 66, 6.9%), progesterone and testosterone (*n* = 13, 1.4%) or progesterone only (*n* = 9, 0.9%). A very small number of participants (*n* = 3, 0.3%) could not be classified into any systemic HRT group following their initial consultation and were therefore excluded from treatment-based analyses, but retained in descriptive statistics. There was no formal ‘no HRT’ control group, as all participants were prescribed some form of treatment post-consultation.

Baseline HRT regime was also recorded, with 48.6% (*n* = 465) of participants not using HRT before entering the clinic. Among those already on treatment, the majority were taking oestrogen and progesterone. Non-systemic HRT users and participants with unclassifiable prescriptions were excluded from HRT-based comparisons, but included in descriptive analyses.

### Measures

#### Depression and suicidality

Depressive symptoms over the past 2 weeks were assessed with the nine-item PHQ-9, a widely used screening tool for depression. Total scores range from 0 to 27, with higher scores indicating greater symptom severity. Commonly used clinical cut-offs are 5 (mild), 10 (moderate), 15 (moderately severe) and 20 (severe depression).^19^ Item 9 of the PHQ-9 assesses suicidal ideation: ‘Thoughts that you would be better off dead or of hurting yourself in some way’. This item was analysed separately and recoded into a binary variable (0 = no ideation; 1–3 = any ideation). Internal consistency in the current sample was high (*α* = 0.82).

#### Menopause-related psychological distress

The MENO-D was used to assess psychological distress specific to menopause. The scale comprises 12 items rated on a 0–4 scale (total score range: 0–48), with higher scores reflecting more severe symptoms across domains such as irritability, paranoia, self-esteem, memory and motivation.^21^ Scores between 20 and 24 indicate mild perimenopausal depression requiring monitoring, 24–32 suggest moderate depression requiring treatment and scores above 32 reflect severe depression requiring treatment. Item 4 specifically asks about ‘Feelings of low self-worth or failure’, with the highest response category indicating thoughts of suicide. This item was analysed separately as a proxy indicator of suicidality. Internal consistency in this sample was acceptable (*α* = 0.80).

### Covariates

Covariates included BMI, smoking status, antidepressant use, baseline HRT status, time between assessments and baseline suicidality. Variables were included in models based on clinical relevance and bivariate correlations. For example, smoking was only included in models where it showed a significant association with the outcome variable. Antidepressant use was retained across all models because of its clinical importance as a proxy for active psychiatric treatment likely to influence mood-related symptoms, regardless of statistical significance.

### Procedure

Clinical data were extracted retrospectively from anonymised records of patients attending routine appointments at a specialist menopause clinic. Participants completed the PHQ-9 and MENO-D as part of standard outcome monitoring. Two timepoints were analysed: baseline (time point 1) and follow-up (time point 2), which occurred on average 107.2 days later (s.d. = 22.6; range 59–338 days). Additional time points (time points 3–5) were not included in the analyses because of high rates of missing data (59% missing at time point 3, 90% at time point 4 and 98% at time point 5), which limited statistical power and model stability. Analyses with >40% missing data on key variables lack confirmatory value,^22^ and recent simulation studies^23^ suggest that although multiple imputation can perform adequately under moderate missingness, its robustness declines beyond 50%, particularly when missingness is not at random or when model assumptions are violated. We therefore determined that including time point 3 would reduce rather than strengthen analytic robustness, and limited analyses to the most complete time points (time points 1 and 2).

To be included in the final analysis, participants had to have complete data for at least one PHQ-9 and one MENO-D at both time points 1 and 2. The final analytic sample ranged from 790 to 898 participants, depending on variable completeness. Item-level analyses (e.g. suicidality, self-worth) allowed for larger sample sizes (up to *n* = 946), as fewer covariates were required.

Data were analysed with mixed-design analyses of variance (ANOVAs) to assess changes in depression, suicidality and menopause-related distress over time. Three-way interaction models (time × HRT regimen × baseline HRT use) were conducted without covariates to preserve model stability and maximise statistical power for exploratory effects. Bonferroni corrections were applied to all follow-up comparisons to adjust for multiple testing and reduce the risk of type 1 error.

## Results

### Sample characteristics

**Fig. 1:**
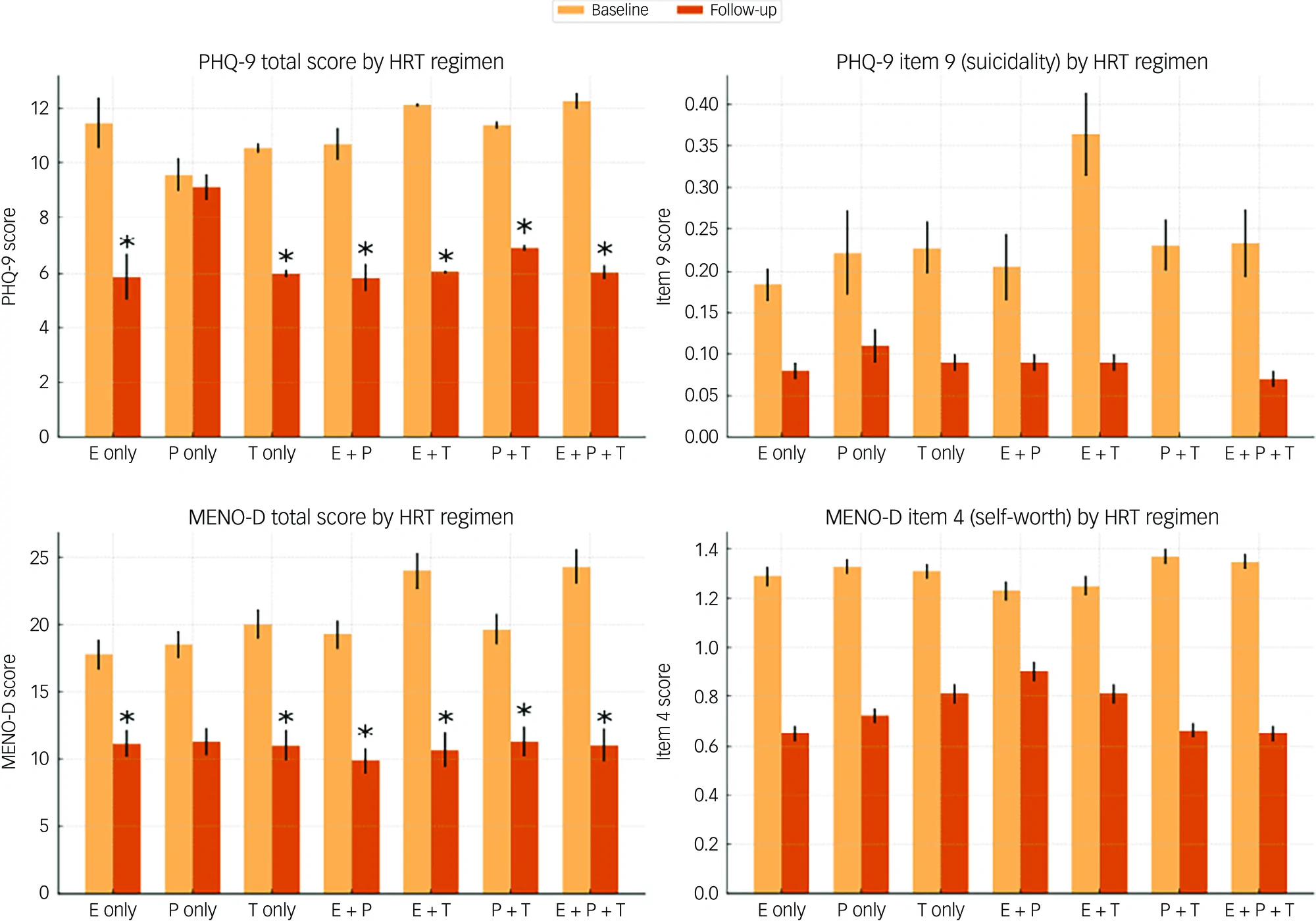
Change in questionnaire scores from baseline to follow-up across four mental health outcomes. Error bars represent ±1 s.e. of the mean. Asterisks indicate significant within-group improvement from time point 1 to 2 (*P* < 0.001). *y*-axis is scaled to observed data to better illustrate meaningful variation. E, oestrogen; MENO-D, Menopause Depression Rating Scale; PHQ-9, Patient Health Questionnaire-9; P, progesterone; T, testosterone.

At baseline, 16.6% of participants (*n* = 159) reported suicidal ideation, which dropped to 6.8% (*n* = 65) at follow-up. Depressive symptoms (PHQ-9 total) decreased from a mean of 11.12 (s.d. = 5.76) to 5.99 (s.d. = 4.77), falling from within the moderate depression range to mild, whereas menopause-related psychological distress (MENO-D total) declined from 19.67 (s.d. = 7.74), just below the threshold for mild perimenopausal depression, to 10.58 (s.d. = 6.92), indicating minimal symptoms well below clinical concern. Self-worth (MENO-D item 4) scores improved from 1.36 to 0.68, and suicidal ideation (PHQ-9 item 9) dropped from 0.23 to 0.08. Figure 1 illustrates the changes in mental health outcomes between baseline and follow-up for the full sample.

All participants received treatment after their initial consultation. Those not on HRT at baseline began treatment thereafter; however, because of the absence of a no-treatment comparator, results must be interpreted with caution regarding natural symptom progression.

### Correlation analyses

PHQ-9 and MENO-D total scores were strongly correlated (*r* = 0.776, *P* < 0.001), indicating substantial overlap in general and menopause-specific depressive symptoms. PHQ-9 item 9 (suicidality) showed weak but significant correlations with both PHQ-9 total (*r* = 0.070, *P* = 0.030) and MENO-D total (*r* = 0.066, *P* = 0.043). MENO-D item 4 (self-worth) was strongly associated with MENO-D total (*r* = 0.704, *P* < 0.001), but not significantly associated with PHQ-9 item 9 (*r* = 0.025). These patterns support the view that suicidality is only partially captured by general depression severity. Lifestyle and clinical variables were also examined, as shown in Table 2.

**Table 2:** Pearson correlation matrix for mental health outcomes and covariates

Variable	PHQ-9 total	MENO-D total	MENO-D item 4 (self-worth)	PHQ-9 item 9 (suicidality)
PHQ-9 total	–	0.776**	0.646**	0.070*
MENO-D total	0.776**	–	0.704**	0.066*
MENO-D item 4 (self-worth)	0.646**	0.704**	–	0.025
PHQ-9 item 9 (suicidality)	0.070*	0.066*	0.025	–
Antidepressant use	0.194**	0.140**	0.104**	0.031
BMI	0.162**	0.197**	0.122**	0.005
Smoking	0.069*	0.061	0.026	0.036

PHQ-9, Patient Health Questionnaire-9; MENO-D, Menopause Depression Rating Scale; BMI, body mass index.

*P < 0.05; **P < 0.01.

### Depression symptoms (PHQ-9 total)

A mixed ANOVA revealed a significant reduction in depressive symptoms over time (*F*(1, 890) = 32.89, *P* < 0.001, *η* ^2^ = 0.036). A significant time × HRT regimen interaction (*F*(7, 890) = 3.56, *P* = 0.001, *η*² = 0.027) indicated that symptom improvement varied by treatment type. There was no significant three-way interaction with baseline HRT use.

Follow-up paired samples *t*-tests showed significant reductions in PHQ-9 scores across all HRT groups, except progesterone-only. The greatest improvements occurred in the oestrogen, progesterone and testosterone group (mean 12.26 to 6.00, *P* < 0.001, *d* = 1.10); oestrogen and testosterone group (mean 12.12 to 6.67, *P* < 0.001, *d* = 1.10); and oestrogen and progesterone group (mean 10.69 to 5.24, *P* < 0.001, *d* = 0.99). Large but comparatively smaller effect size reductions were also observed in the testosterone-only group (mean 10.54 to 5.99, *P* < 0.001, *d* = 0.84), oestrogen-only group (mean 11.45 to 5.88, *P* < 0.001, *d* = 0.96) and progesterone and testosterone group (mean 11.38 to 6.92, *P* = 0.003, *d* = 0.84). The progesterone-only group showed no significant change in PHQ-9 scores (mean 9.56 to 9.11, *P* = 0.356, *d* = 0.27).

### Suicidality (PHQ-9 item 9)

**Fig. 2:**
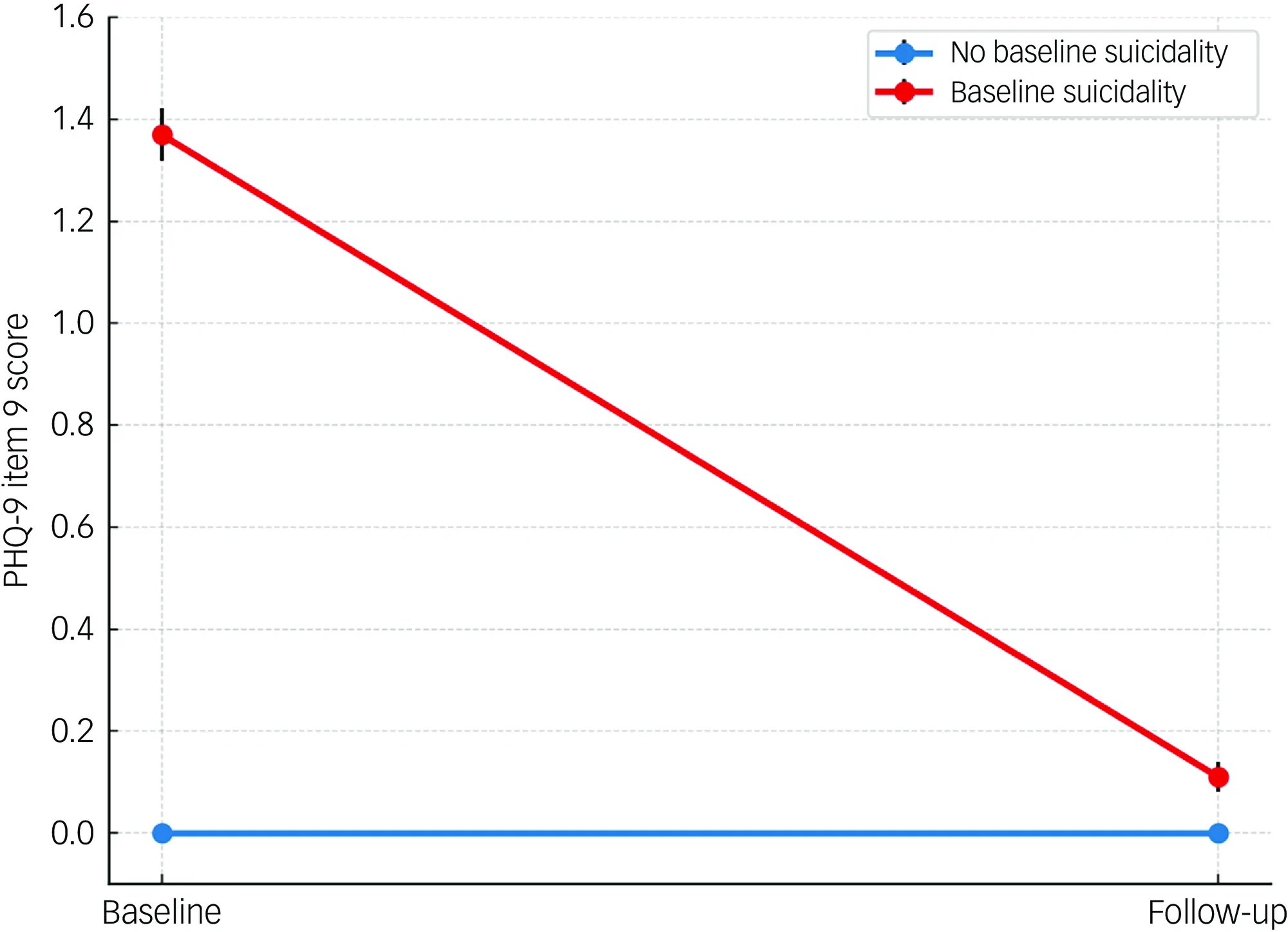
Changes in PHQ-9 item 9 (suicidality) scores by baseline suicidality status. Error bars represent ±1 s.e. of the mean. PHQ-9, Patient Health Questionnaire-9.

There was no main effect of time or time × HRT regimen interaction on suicidality scores. However, a strong time × baseline suicidality interaction emerged (*F*(1, 944) = 867.56, *P* < 0.001, *η*² = 0.479). To further explore this, a paired samples *t*-test was conducted within the subgroup who reported suicidal ideation at baseline (*n* = 160), with mean scores dropping from 1.37 (s.d. = 0.65) to 0.11 (s.d. = 0.38) (*t*(159) = 21.35, *P* < 0.001, *d* = 1.69), as shown in Fig. 2. This effect was not moderated by HRT type or baseline treatment status.

### Menopausal distress (MENO-D total)

Psychological distress significantly decreased over time (*F*(1, 890) = 59.41, *P* < 0.001, *η*² = 0.062). A time × HRT regimen interaction was also significant (*F*(7, 890) = 2.43, *P* = 0.018, *η*² = 0.019), suggesting differential treatment effects. No significant three-way interaction with baseline HRT use was found.

*Post hoc* paired samples *t*-tests showed all groups except progesterone-only experienced significant reductions in distress. The largest improvements were again observed in the oestrogen, progesterone and testosterone group (mean 24.34 to 11.07, *P* < 0.001, *d* = 1.33); oestrogen and testosterone group (mean 24.00 to 10.73, *P* < 0.001, *d* = 1.39); and oestrogen and progesterone group (mean 19.27 to 9.93, *P* < 0.001, *d* = 1.33). Significant reductions were also seen in the testosterone-only group (mean 21.11 to 10.31, *P* < 0.001, *d* = 1.25), oestrogen-only group (mean 21.14 to 10.49, *P* < 0.001, *d* = 1.33) and progesterone and testosterone group (mean 22.38 to 11.08, *P* = 0.004, *d* = 1.16). No significant change was found in the progesterone-only group (mean 19.11 to 18.78, *P* = 0.738, *d* = 0.10).

### Self-worth (MENO-D item 4)

A small but significant main effect of time was found (*F*(1, 890) = 6.09, *P* = 0.014, *η*² = 0.007), with mean self-worth scores improving from 1.29 (s.d. = 0.85) to 0.71 (s.d. = 0.72). However, there were no significant time × HRT regimen or three-way interaction effects, suggesting this improvement was general rather than treatment-specific. Table 3 summarises all the mixed ANOVA models used in the analysis, including the factors tested, covariates included, and statistical outputs.

**Table 3:** Summary of main analysis of variance models by outcome, interaction effects and covariates

Outcome variable	Main effect of time	Time × HRT regimen	Time × baseline HRT use × regimen	Covariates
PHQ-9 total (depression)	*F*(1, 890) = 32.89, *P* < 0.001, *η*² = .036	*F*(7, 890) = 3.56, *P* = 0.001, *η*² = .027	*F*(7, 890) = 1.47, *P* = 0.179	Smoking, antidepressants, time between time points 1 and 2, HRT at baseline, suicidality
PHQ-9 item 9 (suicidality)	*F*(1, 944) = 0.83, *P* = 0.362	*F*(7, 944) = 0.94, *P* = 0.473	*F*(7, 944) = 1.35, *P* = 0.223	BMI, smoking, antidepressants, time between time points 1 and 2, HRT at baseline, suicidality
MENO-D total (menopausal distress)	*F*(1, 890) = 59.41, *P* < 0.001, *η*² = 0.062	*F*(7, 890) = 2.43, *P* = 0.018, *η*² = 0.019	*F*(7, 890) = 0.99, *P* = 0.442	BMI, antidepressants, time between time points 1 and 2, HRT at baseline, suicidality
MENO-D item 4 (self-worth)	*F*(1, 890) = 6.09, *P* = 0.014, *η*² = 0.007	*F*(7, 890) = 0.91, *P* = 0.503	*F*(7, 890) = 1.45, *P* = 0.181	BMI, antidepressants, time between time points 1 and 2, HRT at baseline, suicidality

HRT, hormone replacement therapy; PHQ-9, Patient Health Questionnaire-9; BMI, body mass index; MENO-D, Menopause Depression Rating Scale.

## Discussion

This study aimed to examine the psychological effects of different HRT regimens among menopausal women, focusing on depressive symptoms, suicidality and menopause-related psychological distress. The findings show that mental health symptoms improved over time across most treatment groups, with the most substantial improvements observed in those receiving combined oestrogen, progesterone and testosterone, alongside similarly large effects in the oestrogen and progesterone group and oestrogen and testosterone group. Importantly, suicidality – although only weakly associated with depression scores – declined markedly in those who reported ideation at baseline, reinforcing the need to assess it as a distinct symptom during the menopausal transition.

### Elevated psychological distress and suicidality at entry

The high rate of suicidal ideation at clinical entry – reported by 16.6% of participants – suggests that many women accessing menopause services are doing so in a state of significant psychological distress. This is consistent with broader literature indicating that midlife women, particularly during perimenopause, experience a spike in suicidal thoughts, often under-recognised in healthcare settings.^3,24^ Although it is possible that elevated rates in the present sample partly reflect the nature of the clinic – a specialist service to which individuals may self-refer during acute distress – this does not negate the relevance of the findings. Rather, it highlights the unmet needs and urgency that drive help-seeking among this population, many of whom report long-standing difficulties before referral. The gap between distress prevalence and clinical response may stem from the continued medicalisation or minimisation of menopausal symptoms,^12^ coupled with diagnostic tools that are poorly attuned to menopause-specific experiences.

Although national suicide statistics reflect confirmed deaths rather than ideation,^18^ this study supports the argument that suicidal thinking is more prevalent among menopausal women than previously assumed, and that the menopause itself may represent a high-risk psychological state, not simply a hormonal shift. This aligns with neurobiological theories framing perimenopause as a time of neurochemical sensitivity, particularly in systems involved in mood regulation.^4,25^

### Mental health improvements and hormone regimen differences

Mental health outcomes improved across the study period, but not uniformly. At baseline, PHQ-9 scores across treatment groups ranged from 9.5 to 12.3, indicating that many participants were experiencing moderate depressive symptoms at the point of entry. MENO-D scores similarly suggested clinically meaningful distress. These elevated scores, alongside the 16.6% of participants reporting active suicidal ideation at baseline, underscore the importance of systematic screening for psychological distress and suicidality within menopause care settings. Participants receiving combined oestrogen, progesterone and testosterone experienced the most substantial and consistent reductions in depressive symptoms and psychological distress. These findings support prior work highlighting oestrogen’s stabilising effects on serotonergic and dopaminergic systems,^26^ and further suggest that testosterone may augment mood, motivation and energy: factors critical to psychological recovery.^14,27^ Although oestrogen alone has been shown to improve mild to moderate depression in perimenopausal women,^16,28^ the present study adds to growing evidence that multi-hormonal regimens may be more effective, especially when symptoms are severe or complex.^8,15^ However, given the exploratory nature of these subgroup analyses and the small sample sizes in several HRT regimens, these findings should be interpreted with caution. The strong effect sizes observed across groups were generally comparable, suggesting that different hormone combinations may offer broadly similar levels of psychological benefit rather than clear superiority of one regimen over another. The lack of a non-treatment control group further limits causal attribution and prevents firm conclusions about whether improvements were driven solely by HRT or by non-specific therapeutic effects.

Although improvements were most pronounced in certain HRT regimens, it is also possible that mental health gains reflected a broader combination of factors. Participants may have benefited from holistic menopause care, including psychoeducation, improved sleep, symptom validation or concurrent support for anxiety, relationship or occupational stressors.^29^ Lifestyle changes, antidepressant use or progression through the menopausal transition itself may also have contributed to psychological stabilisation.^30–32^ Given the lack of a non-treatment control group, improvements observed across the study period may reflect both treatment-specific and non-specific effects, and should be interpreted within this broader clinical context.

Conversely, the lack of improvement in the progesterone-only group reflects previous literature highlighting the limited antidepressant properties of progesterone in isolation, as some studies suggest that progesterone may even worsen mood symptoms in susceptible individuals.^16,33^ However, this result should be interpreted cautiously because of the very small sample size in this group (*n* = 9). There is also evidence pointing to potential anxiolytic or stabilising effects in certain contexts,^17,34^ suggesting that progesterone’s psychological impact may vary by symptom type, delivery method or individual sensitivity. Future studies should explore these nuances in more detail.

### Timing of treatment and perimenopausal vulnerability

Around half of the sample was perimenopausal at baseline, which aligns with literature identifying this phase as a window of increased risk for mood disturbance and suicidality.^2^ Hormonal fluctuations during perimenopause may induce mood symptoms that are acute, episodic and reactive; distinct from the more stable presentation seen in major depressive disorder.^21,35^ These symptoms may also be more responsive to hormonal intervention, particularly when treatment is initiated early. The ‘window of opportunity’ hypothesis^36,37^ suggests that initiating HRT near the onset of menopause yields greater benefit across both somatic and psychological domains. Although baseline HRT status did not significantly moderate treatment effects in this study, improvements observed among those initiating treatment during the study period tentatively support the importance of treatment timing. However, conclusions are limited by the lack of detailed data on HRT duration before baseline. Similar patterns have been observed in bone density and cardiovascular outcomes, where earlier intervention appears to enhance biological responsiveness.^38^ However high levels of missingness meant that extended timepoints (e.g. time point 3) were excluded from analysis, as their inclusion would have introduced analytic instability. This decision aligns with established recommendations regarding missing data thresholds.^22,23^

### Suicidality as a distinct symptom dimension

Suicidal ideation declined by over 90% among those who reported it at baseline, with a large effect size, but this improvement was not driven by specific hormone regimens. Instead, the time × baseline suicidality interaction suggests that participants presenting in acute distress improved substantially regardless of treatment type. Although this could partly reflect regression to the mean, the magnitude and consistency of improvement – paired with reductions in depression and self-worth measures – indicates meaningful clinical change for this high-risk subgroup. Notably, suicidality showed only weak associations with total PHQ-9 and MENO-D scores, supporting research that it can occur independently of general depression.^13,20^ This challenges the tendency to treat suicidality as just another symptom of depression and underscores the importance of assessing it directly, particularly in perimenopausal populations, where emotional reactivity and cognitive shifts may heighten risk outside of classical depressive profiles.

The MENO-D item on self-worth, used as a proxy measure, also improved over time, but was not strongly correlated with PHQ-9 suicidality scores. This further supports the need for menopause-sensitive tools that capture suicidality as a specific and nuanced construct rather than relying on indirect indicators.

### Value of menopause-specific measures

Although PHQ-9 and MENO-D total scores were highly correlated, the MENO-D was developed specifically to capture menopause-related psychological features that conventional depression scales may overlook, such as irritability, paranoia and libido changes.^21^ These symptom dimensions may represent a distinct profile of perimenopausal depression,^39,40^ which often manifests more as mood instability than persistent low mood. Although the two scales showed convergence in this sample, the value of the MENO-D lies in its potential to detect cases that would otherwise go unflagged by generic tools. The high correlation observed in this sample may reflect the severity and breadth of symptoms reported by participants, with distress manifesting across both general and menopause-specific domains. This convergence suggests that, in highly symptomatic populations, general depression and menopause-specific mood symptoms may become more tightly intertwined.

Given the unique hormonal and psychological dynamics of perimenopause, further validation of menopause-specific scales is needed. Combining these with general tools may offer a more complete clinical picture, helping to target interventions more effectively.

### Lifestyle factors and treatment response

Higher BMI was significantly associated with worse scores on depression, distress and self-worth, consistent with literature linking elevated BMI to lower mood and self-esteem in women.^37,38^ However, BMI did not significantly moderate the effects of HRT, suggesting that hormone therapy may still be effective across weight categories. Still, there is evidence that higher BMI may alter hormone metabolism or influence pharmacodynamics,^41,42^ which warrants exploration in future dosing studies.

Smoking showed only weak associations with depression and no impact on treatment response. Although smoking has been linked to serotonergic dysregulation and increased depression risk,^43,44^ the small proportion of smokers in this sample (around 4%) may have limited statistical power.

Time between assessments did not moderate outcomes, but was controlled for in all models, to account for variability in treatment exposure duration. This ensured that symptom changes were not confounded by differences in follow-up length.

Although this study was based in a UK specialist clinic, the findings have broader relevance for global mental health systems. In many regions, menopause-related psychological distress and suicidality remain under-recognised, and opportunities for early intervention are often missed. Integrating basic mental health screening into menopause services could offer a scalable strategy for reducing distress and suicide risk across diverse care settings.

### Limitations

Several limitations should be considered when interpreting these findings. First, the study was observational and based on routinely collected clinical data, which limits the ability to infer causality. Without a control group, improvements may partially reflect non-specific factors such as regression to the mean, placebo effects or increased clinical engagement rather than direct effects of HRT. Additionally, participants were recruited from a private specialist menopause clinic, which may limit generalisability to broader populations. Individuals accessing private care may differ in terms of symptom severity, socioeconomic status, health literacy or prior treatment access compared with those using the National Health Service. Replication in more diverse clinical settings is needed to strengthen the applicability of these findings.

Second, HRT dosage and route of administration (e.g. oral versus transdermal) were not captured. This is a notable limitation given that delivery method can influence both hormone levels and mood-related outcomes.^45^ Additionally, duration of prior HRT use was not consistently documented in the clinical data-set and could not be reliably examined in relation to outcomes. Future studies should systematically assess and compare dosage and formulation variables, and explore the role of HRT treatment duration in more depth, given its potential impact on clinical response.

Third, some HRT subgroups – particularly the progesterone-only group – had small sample sizes, which reduced statistical power and limited the generalisability of subgroup comparisons (see ‘HRT classification’). Additional research with more balanced group sizes is needed to confirm the differential effects observed here.

Fourth, although additional time points were available, data for time point 3 were missing for 59% of participants. Including time point 3 would have introduced analytic instability and reduced the robustness of conclusions. Scholars argue that analyses with >40% missing data should not be considered confirmatory,^22^ and studies have shown that multiple imputation techniques perform poorly beyond 50% missingness, particularly when model assumptions may not hold.^23^ As such, we limited analyses to the most complete time points (time points 1 and 2).

Fifth, suicidality was assessed with a single item from each scale. Although PHQ-9 item 9 is widely used in clinical practice, it may not fully capture the nuance and range of suicidal thinking. The use of MENO-D item 4 as a proxy for suicidality added value, but was not designed for this purpose, and should be interpreted cautiously.

Sixth, although lifestyle variables such as alcohol use were collected, inconsistent reporting meant this factor could not be reliably included as a covariate. Given alcohol’s established links to depression and suicidality,^45,46^ its omission is a limitation. Future research should incorporate standardised, high-quality measures of substance use, sleep, physical activity and psychosocial stressors. Additionally, future research could also usefully explore whether changes in other covariates – if collected thoroughly – such as specific symptom domains (e.g. vasomotor symptoms), mediate the relationship between HRT and mental health outcomes. However, caution is warranted because of the potential multicollinearity between overlapping symptom measures within tools like the MENO-D.

Seventh, item-level outcomes (e.g., PHQ-9 item 9, MENO-D item 4) were ordinal, but analysed as continuous variables in ANOVA, which assumes interval-level data. Although this is common in psychological research and enables parametric modelling with covariates, it may obscure categorical nuances. Findings should be interpreted with caution, and future studies may benefit from categorical or non-parametric approaches.

Finally, both the PHQ-9 and MENO-D, although validated for symptom screening, were not specifically designed to track treatment response. This limits precision when interpreting change scores over time. Broader use of clinician-rated outcomes or structured diagnostic interviews may strengthen future evaluations.

### Conclusions

This study provides preliminary evidence that combined HRT – including regimens such as combined oestrogen and progesterone, and combined oestrogen, progesterone and testosterone – may be associated with significant improvements in depressive symptoms and menopause-specific psychological distress among menopausal women. Although suicidality declined over time among those at risk, this effect was not moderated by HRT type, suggesting that broader clinical or psychosocial factors may be at play.

The findings reinforce the importance of assessing suicidality as a distinct outcome during menopause, rather than subsuming it within general depression scores. Standard tools may not fully capture the complexity of mood instability and suicidal ideation during this life stage, highlighting the potential value of menopause-specific instruments like the MENO-D.

Although the observational design limits causal conclusions, the large clinical sample and consistency across measures suggest meaningful psychological improvements associated with multi-hormonal treatment. These results support a more individualised approach to menopause care: one that considers hormonal profile, mental health history and timing of intervention.

Future research should explore the impact of HRT dosage, delivery method and treatment timing by using longitudinal and randomised designs. Direct comparisons of oestrogen-only and oestrogen and testosterone regimens are also warranted, particularly in post-surgical or premature menopause populations, to determine whether testosterone adds incremental benefit beyond oestrogen alone. Further development and validation of menopause-sensitive screening tools are also needed to ensure that suicidality and mood symptoms are accurately identified and addressed. Integrating mental health screening into routine menopause care could improve early detection, guide targeted treatment and ultimately reduce psychological burden during this vulnerable life stage.

## Data Availability

The analytic code used in this study is based on standard statistical procedures conducted in SPSS and has been retained in full by the authors. Although the code is not deposited in a public repository, it is available from the corresponding author upon reasonable request for purposes of result verification or methodological replication. The study used anonymised clinical data collected as part of routine care at a specialist clinic. As such, raw data cannot be publicly shared because of patient confidentiality and ethical restrictions. However, summary materials and full methodological documentation are available from the corresponding author upon reasonable request.
