# Improving detection and treatment of psychological distress during menopause: evidence from a clinical hormone replacement therapy cohort – CORRIGENDUM

**DOI:** 10.1192/bji.2026.10108

**Published:** 2026-08

**Authors:** Olivia Hendriks, Aini Kamal, Pooja Saini, Abigail K. Rose, Daniel Reisel, Louise Newson, Jason C. McIntyre

In the original publication of this article, the author order and author contribution statement were incorrectly stated.

Incorrect author order (as originally published)

Olivia Hendriks, Aini Kamal, Jason C. McIntyre, Abigail K. Rose, Daniel Reisel, Louise Newson, Pooja Saini

Correct author order

Olivia Hendriks, Aini Kamal, Pooja Saini, Abigail K. Rose, Daniel Reisel, Louise Newson, Jason C. McIntyre

Incorrect author contribution statement (as originally published)

“O.H., P.S., J.C.M. and A.K.R. conceived and designed the study. L.N., A.K. and D.R. contributed to study material development and recruitment. A.K. and D.R. collected the data. O.H., P.S., J.C.M. and A.K.R. analysed the data. O.H. led interpretation of study findings. All authors contributed to interpretation of findings. O.H. led the draft of the work. All authors read, commented on and approved the final version of the paper. O.H. is the lead author. P.S. is the senior researcher.”

Correct author contribution statement

“O.H., J.C.M., P.S., and A.K.R. conceived and designed the study. L.N., A.K. and D.R. contributed to study material development and recruitment. A.K. and D.R. collected the data. O.H., J.C.M., P.S., and A.K.R. analysed the data. O.H. led interpretation of study findings. All authors contributed to interpretation of findings. O.H. led the draft of the work. All authors read, commented on and approved the final version of the paper. O.H. is the lead author. J.C.M. is the senior researcher.”

These errors have now been corrected in the published article, and this corrigendum serves as the official notice of correction.
